# The Influence and Mechanism of Curing Methods and Curing Age on the Mechanical Properties of Yellow River Sand Engineered Cementitious Composites

**DOI:** 10.3390/ma17174307

**Published:** 2024-08-30

**Authors:** Kunpeng Zhang, Weijun Wu, Jiahui Fan, Chengfang Yuan

**Affiliations:** 1 Shanghai Highway and Bridge (Group) Co., Ltd., Shanghai 200433, China; 18272948458@163.com (K.Z.); 18538361628@163.com (W.W.); 2 College of Civil Engineering, Zhengzhou University, Zhengzhou 450001, China; 17537183680@163.com

**Keywords:** Yellow River sand (YRS), engineered cementitious composites (ECC), curing method, curing age, mechanical properties, microscopic mechanism

## Abstract

This study investigates the potential use of Yellow River sand (YRS) sourced from the lower reaches of the Yellow River in China as a sustainable and cost-effective substitute for quartz sand in Engineered Cementitious Composites (ECC). With an annual accumulation of approximately 400 million tons in this region, YRS presents a substantial resource. ECC specimens with 100% YRS replacement with quartz sand were subjected to various curing methods: natural, steam, standard, and sprinkler. Extensive mechanical testing including flexural, compressive, uniaxial tensile, and four-point flexural tests was conducted. Additionally, Scanning Electron Microscope (SEM) and Mercury Intrusion Porosimetry (MIP) analyses investigated microscopic mechanisms influencing macroscopic mechanical properties. Finally, the mechanical properties of the YRS-ECC test block after 14 days of standard curing and the traditional sand ECC test block were compared and analyzed. The results indicate that ECC specimens with 100% YRS substitution under natural curing show an optimal ultimate tensile strain of more than 4%, providing the best resistance to the reduction in ultimate flexural load and deflection due to aging. Steam curing enhances flexural and compressive strength, achieving an ultimate flexural load of 5 kN and a maximum deflection of 4.42 mm at 90 days. SEM analysis revealed lower C-S-H gel density under natural curing and higher under steam curing, enhancing fiber pull-out in steam-cured specimens. The MIP tests demonstrated that natural curing had the highest porosity (32.86%) and average pore size (51.69 nm), whereas steam curing resulted in the smallest average pore size, with 44% of pores under 50 nm. Compared with traditional sand, it is found that the ultimate bending load and deflection of YRS-ECC are 5.7% and 9.4% higher than those of traditional sand ECC, respectively, and its ultimate tensile strength and strain are also improved. These findings highlight YRS as a sustainable alternative to natural sand in ECC, with natural curing proving the most effective for superior mechanical performance, including tensile strain, crack resistance, and durability.

## 1. Introduction

Concrete is the most extensively used construction material globally, valued for its versatility, durability, and cost-effectiveness [1]. However, conventional concrete still faces limitations such as low toughness, ductility, and tensile strength, which can lead to corrosion and reduced structural durability [2,3]. To address these shortcomings, researchers have explored various alternative materials including fiber-reinforced concrete [4,5,6], polymer-modified concrete [7,8], self-compacting and lightweight concrete [9,10], high-performance concrete [11], and Engineered Cementitious Composites (ECC) [12]. Among these alternatives, ECC is known for its exceptional ductility, tensile strength, and crack control, which are crucial for enhancing the durability and performance of concrete structures [13]. Foundational research by VC Li on micromechanical modeling established the basis for designing ECC properties [14]. Li and Leung [15] further explored ECC’s behavior under multiple cracking conditions. Yang et al. [16] demonstrated that using high volumes of fly ash improves ECC’s mechanical properties and sustainability. Furthermore, Shanour et al. [17] showed ECC’s enhanced flexural performance in concrete beams. ECC enhances traditional concrete by substituting coarse aggregates with fine materials, typically silica sand, and integrating a low-volume fraction of high-performance fibers like polyvinyl alcohol (PVA) [18]. This modification significantly boosts ECC’s tensile strain capacity, allowing it to achieve ultimate tensile strains ranging from 3% to 7% [19], surpassing those of conventional concrete.

ECC offers several advantages over traditional concrete, including superior toughness and crack resistance, which mitigate issues associated with brittle failure. Its enhanced ductility enables greater deformability under stress, extending the structure’s lifespan and reducing maintenance costs. Additionally, ECC’s ability to self-heal microcracks through autogenous mechanisms further enhances durability [19]. The evolution of construction practices in China, exemplified by landmark projects like the Qinghai–Tibet Railway and the Hong Kong–Zhuhai–Macao Bridge [20,21], has driven the adoption of new materials such as ECC with ultra-high toughness, However, the extensive use of sand in China’s construction sector, totaling over 20 billion tons annually, highlights the growing scarcity of natural sand resources. In response to national imperatives for sustainable and green building [22,23,24,25] materials, researchers have explored alternative local materials, including desert sands [26,27], iron ore tailing aggregates [28], and eco-friendly aggregates like sea sand [29,30] to successfully formulate ECC meeting stress and strain requirements. The substantial sediment deposits of Yellow River sand (YRS) in China’s lower reaches present a significant potential benefit for the construction industry. It is measured by the laser granularity analyzer that particles smaller than 75 μm in YRS constitute approximately 81% of the total weight in the grit chamber, spanning from the lower reaches of the Yellow River to the Jipuyang section. Rich in silica, alumina, and other components, YRS meets ECC’s material selection criteria, demonstrating potential as a sand substitute in ECC formulations [31]. This study aims to conduct a comprehensive series of performance tests to evaluate the feasibility and effectiveness of YRS in practical engineering applications.

Moreover, achieving optimal concrete curing conditions is crucial for ensuring the design strength and durability. Proper curing involves maintaining adequate water and temperature levels to facilitate cement hydration over an extended period [31,32,33]. Factors such as temperature, humidity, and curing duration significantly influence the rate of hydration reactions and the resulting properties of cementitious materials. For instance, high-temperature steam curing can potentially compromise the ductility of ECC [34,35], while insufficient moisture content may affect the long-term strength and durability [36]. Extending the initial water curing period and overall curing duration typically reduces ECC’s deflection and toughness indices, while concurrently enhancing properties such as initial crack load, peak load, compressive strength, and flexural strength [37]. Although most curing durations are standardized to 28 days and often involve a single method, it is essential to explore how Yellow River sand ECC (YRS-ECC) performs across different aging periods [38]. Therefore, it is necessary to explore the performance of YRS-ECC at different curing ages. Various researchers have explored the impact of curing methods on ECC performance. Kim M et al. [39] compared immersion curing with natural curing and observed a significant decrease in ECC’s tensile properties after 3 months of immersion curing, whereas natural curing improved these properties over the same period. Xu et al. [40] conducted tests on ECC’s uniaxial tensile properties at 7, 28, and 60 days under varying relative humidity conditions, revealing that a higher humidity or longer curing age generally increased the initial crack strength and ultimate tensile strength while decreasing tensile strain capacity. Jiang et al. [41] investigated natural curing, standard curing, and early water curing followed by natural curing at different intervals, finding that early soaking curing enhanced compressive and tensile strength more effectively than continuous standard curing. However, different cementitious systems may exhibit varied microstructures and fiber–matrix interface bonding properties depending on curing methods, especially after incorporating YRS into the mix ratio. At present, the research on the influence of different curing methods on the ECC performance of the YRS has not been reported. Therefore, it is essential to examine how different curing methods and aging affect the mechanical properties of YRS-ECC, particularly its ductility.

This study comprehensively explores replacing natural quartz sand with YRS as the fine aggregate in ECC, investigating the impacts of various curing methods and aging stages on the mechanical properties of 100% YRS-ECC. Four curing methods (standard curing, steam curing, sprinkler curing, natural curing) and three curing ages (14 days, 28 days, 90 days) were employed to assess the mechanical properties of YRS-ECC. Additionally, SEM and MIP tests were conducted to analyze the microstructure, hydration degree, and pore characteristics of YRS-ECC, providing a detailed evaluation of the material’s micro-level to macroscopic mechanical properties.

## 2. Materials and Methods

### 2.1. Raw Material

In this study, P.O42.5 ordinary Portland cement produced by Zhengzhou Tianrui Cement Co., Ltd. of Henan Province and grade II fly ash produced by Hengnuo Filter Material Co., Ltd. of Gongyi City, Henan Province, China were used. The fiber employed was polyvinyl alcohol fiber (PVA) produced by Kuraray, Tokyo, Japan, with performance details provided in Table 1. The CQJ-JSS polycarboxylate superplasticizer produced by Chenqi Chemical Technology Co., Ltd. in Shanghai, China is used as a water reducer, and the water reduction rate reaches 26.5%. HPMC-20 hydroxypropyl methyl cellulose produced by Shanghai Chenqi Chemical Technology Co., Ltd.as a thickener is a light-yellow powder with a viscosity grade of 200,000. Ordinary tap water from Zhengzhou, Henan Province, China was used for both mixing and curing.

**Figure 1:**
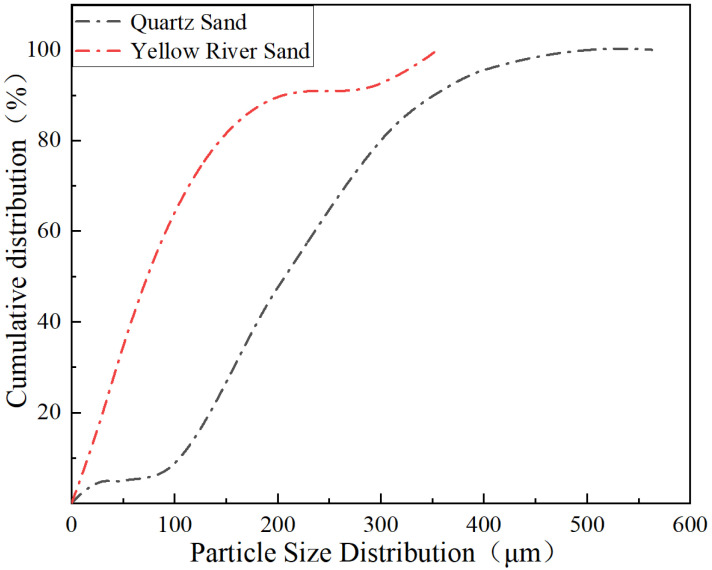
Particle size distribution of YRS and quartz sand.

The YRS used in the test was sourced from the grit chamber of the Yellow River Diversion Project in Puyang City, Henan Province, China. Initially wet, it was directly used as raw material after drying. The quartz sand used in the test was provided by Henan Zhongbang Environmental Protection Technology Co., Ltd., (Zhengzhou, China). The particle size is 80~120 mesh. According to the standard of sand and stone quality and test methods for ordinary concrete [42] (JGJ 52-2006), various technical properties including apparent density, bulk density, saturated surface dry water absorption rate, and specific surface area of quartz sand and YRS were tested and are detailed in Table 2. The particle size of the two kinds of sand was analyzed by the Malvern Mastersizer-2000 laser particle size analyzer. The YRS particle size range is 0 to 355 μm, and the quartz sand particle size range is 0–565 μm, as depicted in Figure 1. Among them, the D10 of quartz sand is 105.7 (the particles below 105.7 μm account for 10%, the same below), D25 is 150.4, D50 is 204.9, D75 is 249.8, and D90 is 348.5. D10 of YRS is 10.5, D25 is 38.1, D50 is 69.0, D75 is 110.2, and D90 is 157.4. Overall, the D [3,2] (surface area average particle size, the same as below) of quartz sand is 82.2 μm, and the D [4,3] (volume average particle size, the same as below) is 214.5 μm; the D [3,2] of the YRS is 13.8 μm, and the D [4,3] is 79.2 μm. The particle size of the YRS is finer than that of quartz sand, and the particle size distribution is more concentrated.

### 2.2. Mix Proportion

In the early stage, a large number of experimental studies have been carried out on the mix ratio of ECC prepared by quartz sand, and PVA-ECC with strain-hardening characteristics and uniaxial tensile strain of more than 3% has been successfully prepared. The coordination at a 100% substitution rate is shown in Table 3.

### 2.3. Specimen Preparation

**Figure 2:**
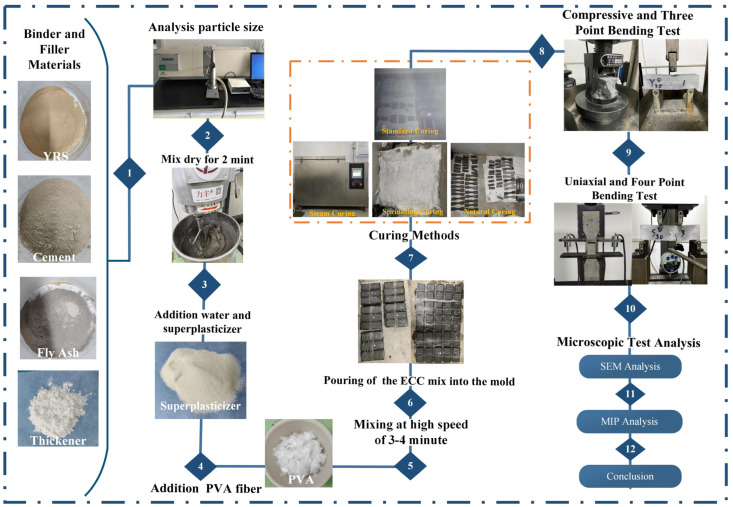
Experimental process and methodology of the research.

The ECC specimens were prepared using a Lifeng planetary strong mixer. The specific pouring process was as follows: Firstly, all cement, fly ash, and sand were added sequentially and dry mixed at low speed for 2 min. Secondly, water, water-reducing agent, and a thickening agent were added, followed by medium-speed wet mixing for 2 min. Thirdly, PVA fiber was introduced, and the mixture underwent high-speed stirring for 3 min to ensure uniform fiber distribution. Once the ECC slurry was prepared, it was poured into the test mold twice. The slurry was then compacted using a shaking table and vibrating rod to achieve density, and the surface was covered with a preservative film to prevent water evaporation. After allowing the specimens to stand at room temperature for 48 h, the molds were removed, and the specimens were transferred to different curing environments for further curing. The experimental process and method of the study are shown in Figure 2.

### 2.4. Curing Method

**Figure 3:**
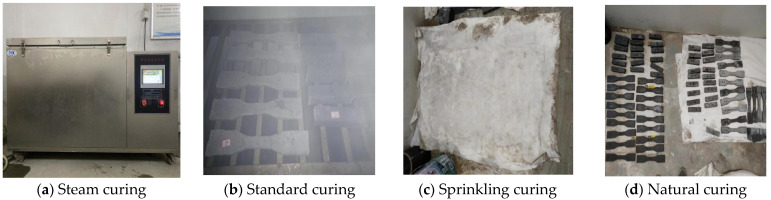
YRS-ECC specimens under different curing methods.

In this experiment, four distinct curing methods were implemented to assess their impact on the ECC specimens: steam curing (Z), standard curing (B), sprinkling curing (S), and natural curing (K), as illustrated in Figure 3. Each method was applied across three different curing ages: 14 days, 28 days, and 90 days. For steam curing (Z), specimens were initially placed in a steam curing box for 3 days post-demolding, followed by transfer to a controlled environment at 20 °C ± 2 °C and 50% relative humidity until reaching the specified curing ages. Standard curing (B) involved immediate placement of specimens post-demolding in a room maintained at 20 °C ± 2 °C with relative humidity exceeding 95%, followed by curing to 14 d, 28 d, and 90 d. Sprinkling curing (S) required specimens to be placed indoors post-demolding and covered with geotextile, receiving morning and evening sprinkling sessions. They were then cured until reaching 14 d, 28 d, and 90 d ages. Lastly, natural curing (K) involved placing specimens in a room with a temperature of 20 °C ± 2 °C and 50% relative humidity immediately after demolding, continuing until 14 d, 28 d, and 90 d ages were achieved. Throughout the study, ‘Z, B, S, and K’ denoted the curing methods, followed by the number indicating the curing age. For example, ‘Z14’ represents the Yellow River sand ECC under 14 d steam curing conditions. In addition, the test also set up traditional sand ECC under standard curing for 28 days, expressed as “B’28”.

### 2.5. Test Method

In this experiment, the effects of different curing methods and curing ages on YRS-ECC were analyzed by compressive and flexural test, uniaxial tensile test and four-point bending test, and the mechanical properties of YRS-ECC and quartz sand ECC were compared and analyzed. Table 4 shows the grouping of the test, including the size and number of each test and specimen.

#### 2.5.1. Compressive and Flexural Tests

**Figure 4:**
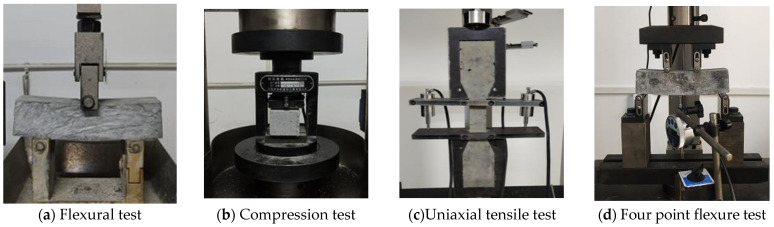
Test setup and instrumentation.

The tests were conducted in accordance with the standard ‘Cement mortar strength test method’ (GB/T 17671-2021) [43]. Cuboid specimens measuring 160 mm × 40 mm × 40 mm were selected, with three specimens tested per group. The testing was performed using the Jinan Tianchen cement flexural and compressive integrated testing machine. During the flexural tests, the forming surface of the specimen was positioned outward, and the loading speed was set at (50 ± 10) N/s for the flexural test, as depicted in Figure 4a. Following the flexural test failure, the compressive strength was measured on the half-cut prism specimens. The side of the specimen was chosen as the compression surface, with a loading area of 40 mm × 40 mm and a loading speed of (2400 ± 200) N/s, as illustrated in Figure 4b.

#### 2.5.2. Uniaxial Tensile Test

The uniaxial tensile tests were conducted following the specifications outlined in ‘JCT 2461-2018’ High Ductility Fiber Reinforced Cement-Based Composite Material Mechanical Properties Test Method [44]. Specimens sized at 330 mm × 60 mm × 13 mm were prepared, with 6 specimens tested per group. The tests were performed using the WDW-20 microcomputer-controlled electronic universal testing machine from Jinan Hengle Xingke Instrument Co., Ltd. (Jinan, China), equipped with the DN3816N static stress and strain test and analysis system from Jiangsu Donghua Test Company (Taizhou, China). The testing employed a displacement control loading method at a rate of 0.5 mm/min, as depicted in Figure 4c. This setup ensured consistent and controlled loading conditions throughout the uniaxial tensile tests to accurately measure the mechanical properties of the specimens.

#### 2.5.3. Four Point Flexural Test

Due to the two-dimensional distribution of fibers in thin plate specimens, which differs from three-dimensional distributions, this study utilized a 160 mm × 40 mm × 40 mm cuboid for the four-point flexural test, with three specimens tested in each group. The tests were conducted on the WDW-20 microcomputer-controlled electronic universal testing machine manufactured by Jinan Hengle Xingke Instrument Co., Ltd., following the guidelines outlined in the specification ‘DBJ61/T 112-2021’ High Ductile Concrete Application Technical Specification [45]. During testing, a dial gauge was positioned beneath the specimen’s mid-span load and connected to the computer for data acquisition. Displacement control loading was employed, with a loading rate set at 0.2 mm/min, as illustrated in Figure 4d. This setup ensured precise control over the loading conditions, facilitating accurate measurement and analysis of the four-point flexural properties of the specimens.

#### 2.5.4. Flexural Toughness Evaluation

This study adopts the flexural toughness evaluation method as outlined by Dai Jie [46], utilizing a trabecular specimen sized at 160 mm × 40 mm × 40 mm. In this method, the corresponding equivalent flexural strengths *feq.0*, *feq.1*, and *feq.2* are employed to characterize the material’s elasticity, limit, and post-peak performance, respectively. These parameters provide a comprehensive assessment of the flexural toughness of the material under study.

(1) Equivalent flexural strength *f_eq.u_* based on peak load, is calculated as Equation (1):

(1) $$f_{eq.u}=\frac{\Omega_{u}L}{bh^{2}\delta u}$$

where *f*_e*q.u*_ is the equivalent bending strength (N/mm^2^); Ω*_u_* is the area under the load–deflection curve when the mid-span deflection is *δ_u_* (N.mm); *δ_u_* is the corresponding deflection value when the load drops to u times the peak load (mm); *b* and *h* are the width and height of the cross-section of the specimen, respectively (mm); and *L* is the span of the specimen (mm).

(2) Based on the CSCE13 2009 [47], improved flexural toughness ratio *R*_e.u_ is calculated as Equation (2):

(2) $$R_{e.u}=\frac{f_{eq.u}}{f_{eq.0}}$$

where *R*_e.u_ is the bending toughness ratio, which represents the ratio of the equivalent bending strength of the specimen at a certain stage to the bending strength of the initial crack, and reflects the fiber bridging effect and the bending performance of ECC material after cracking from the two angles of deformation and energy dissipation; *f*_eq.0_ is the initial cracking bending strength; and *f*_eq.u_ is the equivalent bending strength when u takes 1 and 0.85.

#### 2.5.5. SEM Experiments

The cement hydration products, local crack sections, and fiber pull-out damage in the specimens were observed using the Field Emission Scanning Electron Microscope (Hitachi Ltd., Tokyo, Japan). This equipment offers an image resolution of 0.8 nm and operates within an acceleration voltage range of 0.5 to 30 kV. The microscope provides a magnification capability ranging from 20 to 1,000,000 times, enabling detailed examination and analysis of the microstructural features and damage mechanisms within the specimens.

#### 2.5.6. MIP Experiments

The mercury intrusion test in this study utilized the American Mac AutoPore IV 9500 produced by McMurray Tick (Shanghai) Instrument Co., Ltd. (Shanghai, China). This equipment is capable of generating a maximum pressure of 60,000 psia and can measure pore sizes within the range of 3.0 nm to 1000 μm. The sample size used for testing was approximately controlled at 20 mm^3^, ensuring consistent and accurate analysis of pore characteristics such as pore volume and size distribution within the specimens

## 3. Results and Discussion

### 3.1. Effect of Curing Methods on Mechanical Properties

#### 3.1.1. Compressive and Flexural Strength

**Figure 5:**
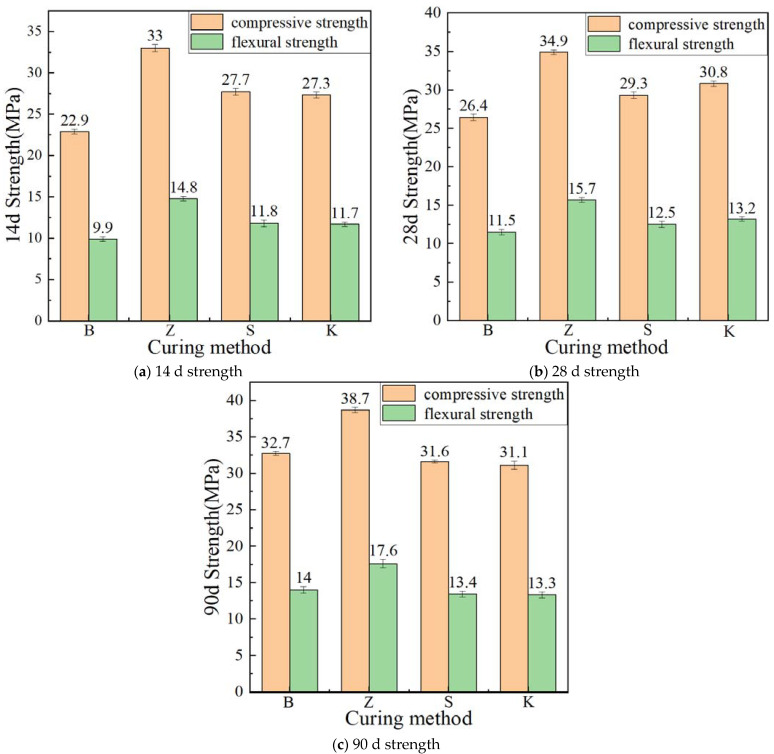
Effect of curing methods on compressive and flexural strength of YRS-ECC.

The percentage change in compressive strength with different curing methods is shown in Table 5. Based on the strength under standard curing for 14, 28, and 90 days, the impact of various curing methods on the flexural strength of YRS ECC is illustrated in Figure 5. At a curing age of 14 days, ECC specimens subjected to steam curing exhibited the highest compressive strength of 33 MPa and flexural strength of 14.8 MPa. This enhancement can be attributed to the elevated temperature during steam curing, which accelerates cement hydration and promotes early secondary hydration of fly ash, leading to increased formation of C-S-H gels. In contrast, ECC specimens cured under standard conditions showed the lowest compressive strength of 22.9 MPa and flexural strength of 9.9 MPa, likely due to the slower hydration process without the immediate benefits of continuous and stable moisture supply. Under sprinkler curing, ECC exhibited compressive and flexural strengths of 27.7 MPa and 11.8 MPa, respectively, slightly lower than those under steam curing. This method, despite providing intermittent water supply, still facilitated sufficient hydration during the early stages comparable to immersion curing. Natural curing resulted in compressive and flexural strengths of 27.3 MPa and 11.7 MPa, respectively, showing only a marginal decrease compared to sprinkler curing, indicating adequate internal water content for effective cement hydration at 14 days.

At 28 days of curing, ECC specimens under steam curing maintained the highest compressive strength of 34.9 MPa and flexural strength of 15.7 MPa. Natural curing showed improved strength with compressive and flexural values of 30.8 MPa and 13.2 MPa, respectively, surpassing those under sprinkler curing. Standard curing continued to exhibit the lowest strengths at 26.4 MPa compressive and 11.5 MPa flexural, underscoring the importance of consistent water vapor supply for long-term hydration.

By 90 days, steam curing sustained its superiority with ECC achieving 38.7 MPa compressive and 17.6 MPa flexural strengths. Standard curing improved significantly to 32.7 MPa compressive and 14.0 MPa flexural strengths, surpassing those of sprinkler and natural curing methods at this age. Watering curing and natural curing showed comparable strengths at 31.6 MPa and 31.1 MPa compressive and 13.4 MPa and 13.3 MPa flexural, respectively, highlighting their similar effectiveness in providing necessary moisture for extended hydration periods.

#### 3.1.2. Uniaxial Tensile Property

**Figure 6:**
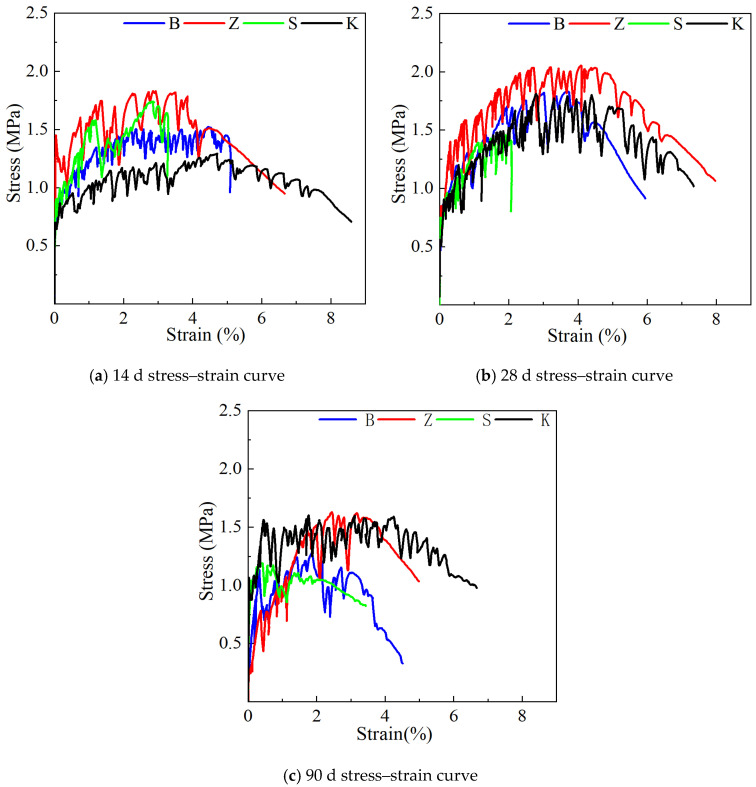
Effect of curing methods on uniaxial tensile properties of YRS-ECC.

Figure 6 illustrates the influence of different curing methods on the uniaxial tensile properties of YRS-ECC. At a curing age of 14 days, ECC specimens cured naturally exhibited the highest maximum tensile strain of 4.70%, albeit with the lowest ultimate tensile strength at only 1.3 MPa. This outcome is attributed to the reliance solely on internal water for hydration in natural curing, which supports better fiber pull-out but results in lower matrix strength. Conversely, specimens cured under steam curing showed an ultimate tensile strength of 1.82 MPa, 1.4 times higher than natural curing, due to accelerated cement hydration and the pozzolanic effects of fly ash. However, steam curing reduced ductility, evident from the strain of 3.50% and notable curve fluctuations. Under sprinkler curing, ECC achieved an ultimate tensile strength of 1.74 MPa with a tensile strain of 2.82%, while standard curing yielded a tensile strength of 1.54 MPa and strain of 4.47%. The higher water supply in sprinkler curing enhanced matrix strength but reduced specimen ductility compared to standard curing.

At 28 days of curing, ECC specimens under steam curing maintained the highest ultimate tensile strength of 2.05 MPa. Standard curing, natural curing, and sprinkler curing yielded tensile strengths of 1.83 MPa, 1.79 MPa, and 1.46 MPa, respectively, with strains close to 4.37% for natural curing and 3.71% for standard curing. Steam curing facilitated robust pozzolanic activity and interface bonding, enhancing ductility despite the strain reducing slightly to 4.03%. Conversely, watering curing exhibited lower ductility (ultimate tensile strain of 1.91%) due to higher matrix density inhibiting complete fiber pull-out.

By 90 days, ECC specimens under natural curing achieved the highest tensile strain of 4.25%, followed by steam curing (3.18%), standard curing (1.86%), and sprinkler curing (0.73%). The ultimate tensile strength decreased from steam curing (1.62 MPa) to natural curing (1.59 MPa), standard curing (1.27 MPa), and sprinkler curing (1.17 MPa). Standard and sprinkler curing failed to meet the 3% strain requirement due to prolonged water provision, leading to reduced pseudo-strain and strain hardening, limiting ultimate tensile strain.

These findings underscore the significant impact of curing methods on the mechanical performance of YRS-ECC, highlighting trade-offs between the strength and ductility based on the hydration conditions and water availability during curing.

#### 3.1.3. Four-Point Bending Test

**Figure 7:**
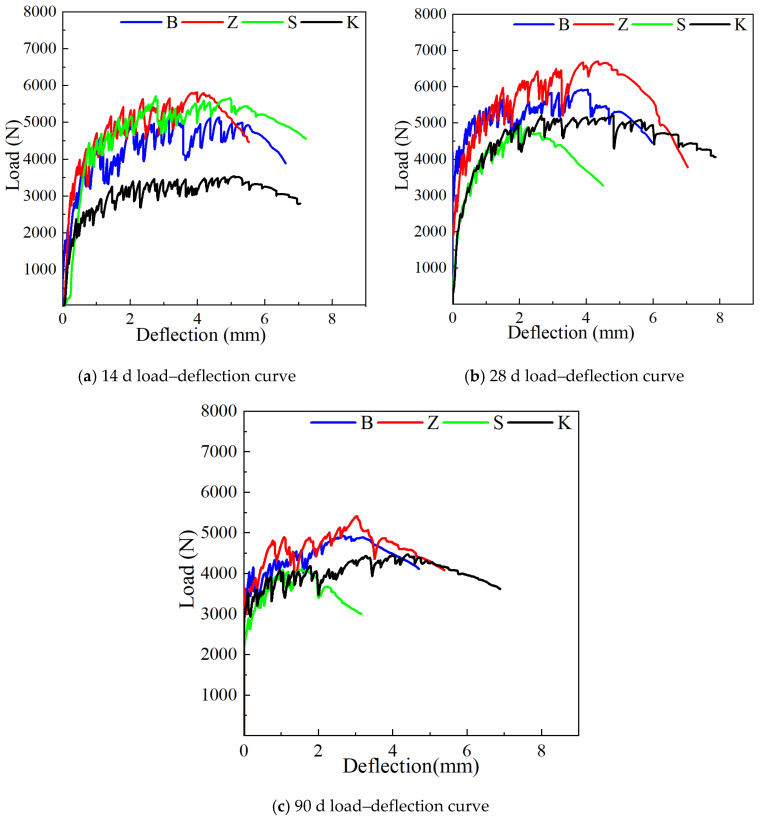
Effect of curing methods on four-point flexural properties of YRS-ECC at different ages.

Figure 7 illustrates the impact of various curing methods on the four-point flexural performance of YRS ECC. At the ages of 14 days, 28 days, and 90 days, the ECC specimens under steam curing consistently exhibit the highest initial crack load, while those cured naturally consistently display the lowest initial crack load. Standard curing and sprinkler curing fall between these extremes

At 14 days, ECC specimens under natural curing show the lowest ultimate flexural load at 3534.01 N but achieve the maximum deflection of 5.1 mm. This is attributed to lower matrix strength facilitating fiber pull-out and enhancing pseudo-strain hardening. Steam curing, sprinkler curing, and standard curing yield similar ultimate flexural loads—5817.03 N, 5708.99 N, and 5132.65 N—with corresponding deflection values of 4.76 mm, 2.77 mm, and 3.99 mm, respectively. At 28 days, the ultimate flexural load ranks highest to lowest as follows: steam curing at 6693.04 N, standard curing at 5917.73 N, natural curing at 5200.61 N, and sprinkler curing at 4917.41 N. However, the order of ultimate flexural deflection slightly differs, with natural curing exhibiting the highest deflection at 4.82 mm, followed by steam curing at 4.36 mm, standard curing at 4.04 mm, and sprinkler curing at 2.04 mm.

However, at the age is 90 days, ECC specimens under steam curing and standard curing demonstrate similar ultimate flexural loads—5004.82 N and 4912.64 N—and deflections of 3.05 mm and 2.87 mm, respectively. In contrast, specimens under natural curing exhibit a deflection of 4.42 mm and a load of 4473.02 N. Sprinkler curing results in the smallest deflection at 1.56 mm and the lowest ultimate load at 4108.34 N due to varying external water supply. The load–deflection curve further illustrates the extended strain-hardening stage under natural curing conditions compared to sprinkler curing, which limits the increase in ultimate load. These findings highlight the significant influence of curing methods on the flexural performance of YRS-ECC, emphasizing trade-offs between the initial crack resistance, ultimate load capacity, and ductility based on the hydration conditions and water availability during curing. The load–deflection curve (shown in Figure 7c) further confirms this point. The strain-hardening stage under natural curing conditions is significantly longer than that under watering curing conditions, which indicates that a shorter strain-hardening process may lead to a limited increase in ultimate load, thus explaining the phenomenon that the ultimate load of the specimen under watering curing is lower.

#### 3.1.4. Flexural Toughness

**Figure 8:**
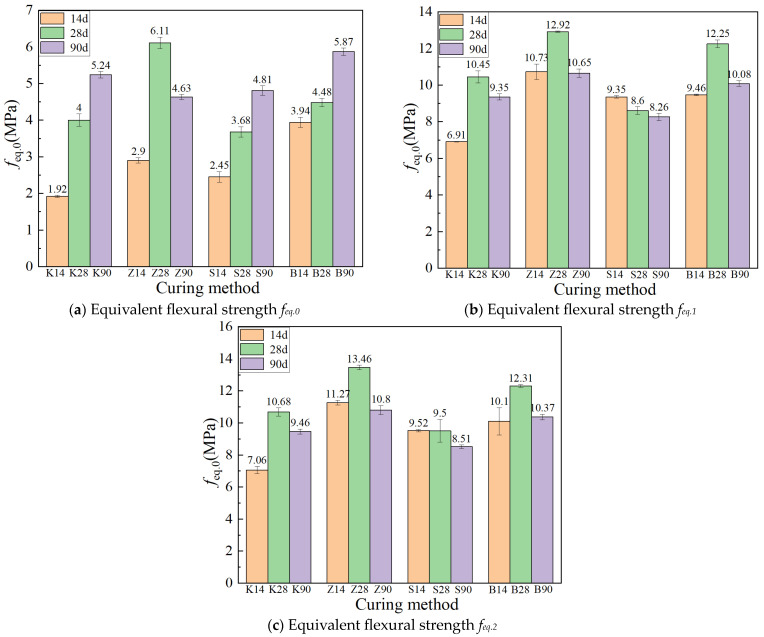
Equivalent flexural strength under different curing methods.

The equivalent flexural moment strength under different curing methods is shown in Figure 8. As the age increases from 14 days to 28 days, the peak equivalent flexural strength *f_eq.1_* and the descending equivalent flexural strength *f_eq.2_* (*μ* = 0.85) of the specimens continue to increase under the other three curing methods except for sprinkler curing. When the age increases from 28 days to 90 days, the development trend in the equivalent flexural strength *f_eq.2_* in the descending section is similar to that of *f_eq.1_*. For instance, the specimens under standard curing and steam curing conditions have higher ultimate flexural loads, but the decrease of *f_eq.1_* under these two curing methods is also larger, decreasing by 17.7% and 17.6%, respectively. In contrast, the decrease of *f_eq.1_* under natural curing conditions is small, only 10.5%, indicating that the specimens under natural curing show higher stability in toughness. This result suggests that natural curing may be more conducive to maintaining the material’s toughness during long-term curing.

**Figure 9:**
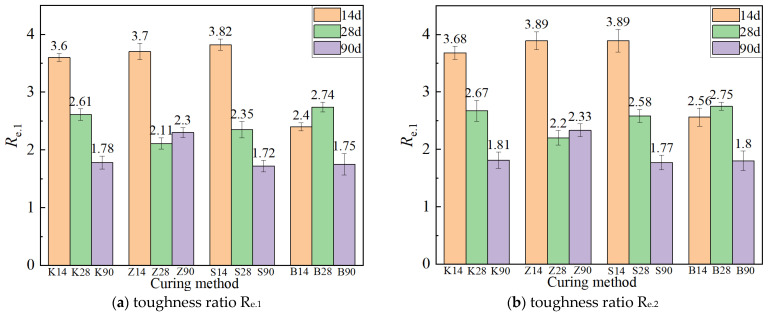
Toughness ratio under different curing methods.

The toughness ratios Re.1 and Re.2 under different curing methods decrease with increasing age (Figure 9). This is because at a longer age, the matrix strength is higher, the bonding ability between the fiber and the interface is stronger, the number of fiber fractures when pulled out is greater than at shorter ages, and the deformation ability is weakened, resulting in a decrease in toughness. At the age of 90 days, the order of toughness ratios (Re.1 and Re.2) from high to low is steam curing, natural curing, standard curing, and sprinkler curing, consistent with the results of the four-point flexural test. This indicates that toughness is a comprehensive index for evaluating energy dissipation capacity and deformation capacity. The ultimate flexural load of ECC under steam curing is high, and the energy absorbed by cracking is substantial. Although the ultimate flexural load of ECC under natural curing is not high, the deformation ability is strong and the ultimate flexural deflection is the largest. Therefore, ECC under steam curing and natural curing are superior in toughness compared to ECC under standard curing and sprinkler curing.

### 3.2. Effect of Curing Age on Mechanical Properties

#### 3.2.1. Compressive and Flexural Strength

**Figure 10:**
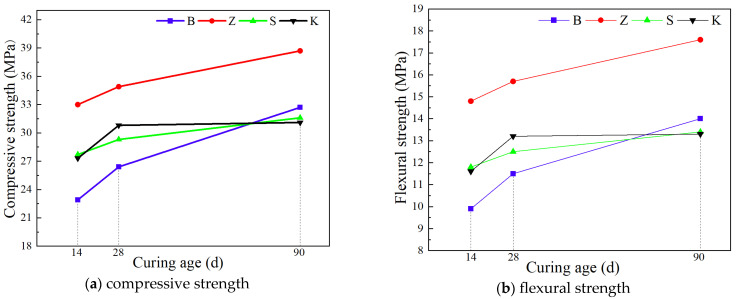
Effect of curing age on the flexural strength of YRS-ECC.

In this study, the 14 d age under each curing method is used as a benchmark to measure the influence of the growth of the curing age on the compressive strength, and the growth rate is calculated. The percentage change in compressive strength under different curing times is shown in Table 6. The effect of different curing ages on the compressive strength of the YRS-ECC is shown in Figure 10.

When the curing age increases from 14 days to 28 days, the strength of YRS-ECC under standard curing increases the fastest, with compressive and flexural strength increasing by 15.28% and 16.16%, respectively. Under natural curing, the compressive and flexural strength increase by 12.82% and 13.80%, respectively. In contrast, the strength growth rate under steam curing is relatively slow, with compressive and flexural strength increasing by 5.76% and 6.08%, respectively. This is because, after three days of high-temperature and high-humidity steam curing, most of the cementitious materials in the specimen have completed the hydration process, resulting in limited subsequent strength growth. The strength growth rate of ECC under sprinkler curing is similar to that under steam curing, with compressive and flexural strength increasing by 5.78% and 5.93%, respectively. This is likely because most of the cement in the specimen has already hydrated by the age of 14 days, limiting subsequent strength growth.

When the curing age increases from 28 days to 90 days, the strength of YRS-ECC under standard curing continues to grow at the highest rate, with compressive and flexural strength increasing by 23.86% and 21.74%, respectively. Under natural curing conditions, the strength growth of ECC is almost stagnant, with the strength at 90 days being only slightly higher than that at 28 days. The compressive and flexural strength of the specimens under steam curing increased by 10.89% and 12.10%, respectively. The compressive and flexural strength growth rates of the specimens under sprinkler curing are 7.85% and 7.20%, respectively, which are lower than the growth rates of the specimens under standard curing.

#### 3.2.2. Uniaxial Tensile Property

**Figure 11:**
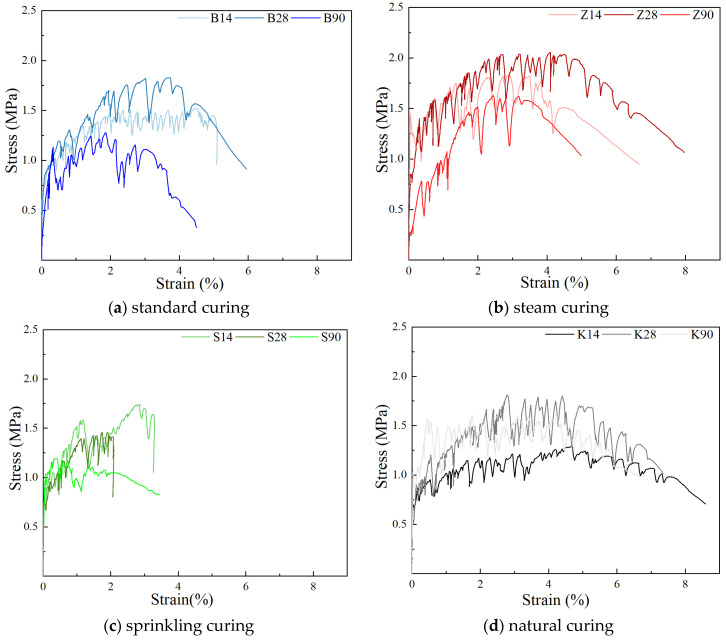
Effect of curing age on uniaxial tensile properties of YRS-ECC.

The effect of curing age on the uniaxial tensile properties of YRS ECC is shown in Figure 11. With the increase in curing age, the specimens under different curing methods show different trends in tensile properties.

When the curing age increases from 14 days to 28 days, the ultimate tensile strength of the specimen under natural curing shows the fastest increase, reaching 37.7%. This rapid increase is attributed to the lack of water in the specimen under natural curing, resulting in low strength at 14 days. However, by 28 days, the specimen is still in the early age stage, and a significant amount of cement hydration occurs due to the internal water of the specimen. The rapid growth in strength under natural curing leads to a slight decrease in ultimate tensile strain by 7.4%. For specimens under steam curing and standard curing, the ultimate tensile strength increases by 12.6% and 19.6%, respectively. Interestingly, the ultimate tensile strain of the specimen under steam curing increases by 15.1%, while it decreases by 17% under standard curing. This is because high temperatures accelerate the secondary hydration of mineral admixtures, consuming more Ca(OH)_2_ and weakening the chemical bonding between fibers and the matrix, thus allowing most fibers to be smoothly pulled out. Conversely, the ultimate tensile strength and tensile strain of specimens under sprinkler curing show a downward trend, decreasing by 19.2% and 32.5%, respectively.

When the curing age increases from 28 days to 90 days, the tensile strain of the YRS-ECC specimen under natural curing remains stable at around 4%, although the ultimate tensile strength decreases by about 10%. The ultimate tensile strength and tensile strain of specimens under steam curing decrease by about 26%, but the strain remains greater than 3%. The ductility and strength of specimens under standard curing and sprinkler curing decrease more significantly, with tensile strain decreasing by 49.9% and 61.2%, respectively, and ultimate tensile strength decreasing by 43.0% and 19.9%, respectively. This reduction is because the sufficient water provided by these two curing methods increases the chemical bonding strength at the fiber–matrix interface, making it difficult for most fibers to be pulled out, which results in the specimens failing to crack effectively.

#### 3.2.3. Four-Point Bending Test

**Figure 12:**
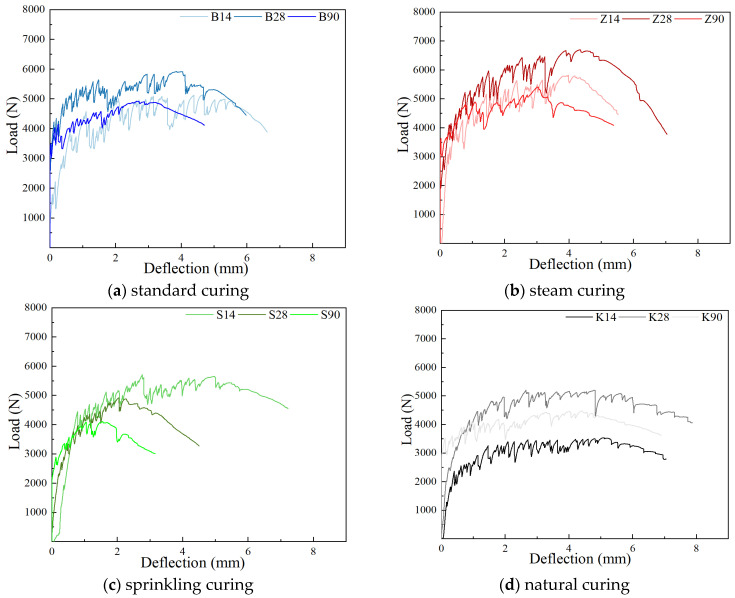
Effect of curing age on four-point flexural performance of YRS-ECC.

The effect of curing age on the uniaxial tensile properties of YRS-ECC is shown in Figure 12.

Effect of curing age on initial crack load. When the curing age increases from 14 days to 28 days, the initial crack load of YRS-ECC under natural curing, standard curing, sprinkler curing, and steam curing increases by 103.4%, 55.1%, 24.6%, and 23.9%, respectively. From 28 days to 90 days, the initial crack load under these four curing methods continues to increase, although the growth rate slows down. Under natural curing, standard curing, sprinkler curing, and steam curing, the initial crack load increases by 20.3%, 21.6%, 17.8%, and 8.8%, respectively. This trend indicates that with the extension of the curing age, the growth of the initial cracking load of specimens under different curing methods tends to stabilize.

Effect of curing age on ultimate flexural load and ultimate flexural deflection. When the curing age increases from 14 days to 28 days, the load–deflection curve of the YRS-ECC specimen under natural curing shows a significant increase (Figure 12d), corresponding to a substantial rise in ultimate flexural strength by 47.1% and a slight decrease in deflection by 5.5%. The ultimate flexural load increase rates of specimens under steam curing and standard curing are similar, at 15.1% and 15.3%, respectively. However, the ultimate flexural deflection of the former continues to increase by 9.3%, while the deflection of the latter decreases by 15.1%, consistent with the uniaxial tensile test results. The load–deflection curve of the specimen under sprinkler curing shows a “shrinkage” phenomenon, with ultimate flexural strength and ultimate flexural deflection decreasing by 13.9% and 26.4%, respectively. This indicates that sufficient water at an early age is not conducive to the multi-crack steady-state cracking of the YRS-ECC.

**Figure 13:**
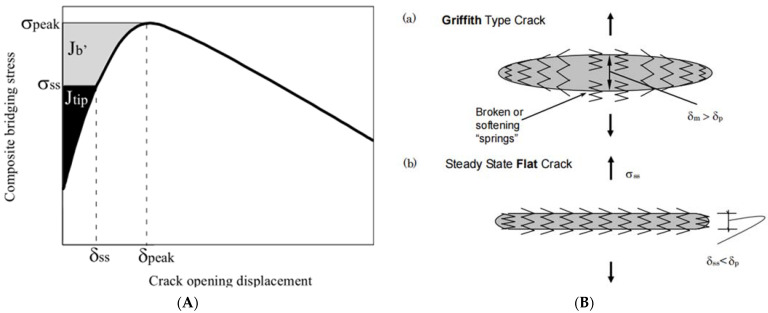
Crack propagation dynamics and energy release criterion diagram. (**A**) Pseudo-strain-hardening energy criterion [50,51]. (**B**) Two different forms of crack propagation [51].

When the curing age changes from 28 days to 90 days, the load–deflection curves of YRS-ECC under the four curing methods show different degrees of “shrinkage.” The ultimate flexural strength of the specimens under steam curing, standard curing, sprinkler curing, and natural curing decreases by 25.2%, 16.8%, 16.6%, and 13.9%, respectively. The ultimate flexural deflection decreases by 30.0%, 28.9%, 23.5%, and 8.3%, respectively. Under natural curing, the curve of the specimen shows the smallest degree of “shrinkage” and maintains the highest ultimate flexural deflection of 4.42 mm. This is because achieving high ductility in ECC requires satisfying the strength criterion and the full energy criterion: the maximum complementary energy J_b_’ must be greater than the crack tip expansion strength J’_tip_ (crack tip expansion strength) to achieve multi-crack cracking (Figure 13A). Kanda pointed out that the pseudo-strain-hardening phenomenon is divided into two types: saturated and unsaturated [48]. The degree of saturation of the crack depends on the ratio of J_b_’ to J’_tip_. When the supplemental energy is too small, the crack propagates around, and the crack tip will produce a strain softening zone. The crack propagation form is similar to the Griffith crack propagation process, resulting in unsaturated strain hardening. When the complementary energy is large, the crack will expand stably, and the fiber at the fracture can withstand the continuously increasing tensile load, and the stress at the crack is transmitted back to the matrix, and new cracks are generated to achieve saturated strain hardening (Figure 13B). J_b_′/J′_tip_ = 3 is the boundary value of saturated multiple cracking [49]. With the increase in curing age, the matrix strength increases continuously. The load–deflection curves indicate that the starting section of the curve for all four curing methods gradually moves to the left, reducing the ratio of J_b_’ to J’_tip_, this is reflected in the decrease in the saturation degree of cracks and a reduction in ductility.

### 3.3. Material Microstructure Analysis

#### 3.3.1. SEM Experiments

**Figure 14:**
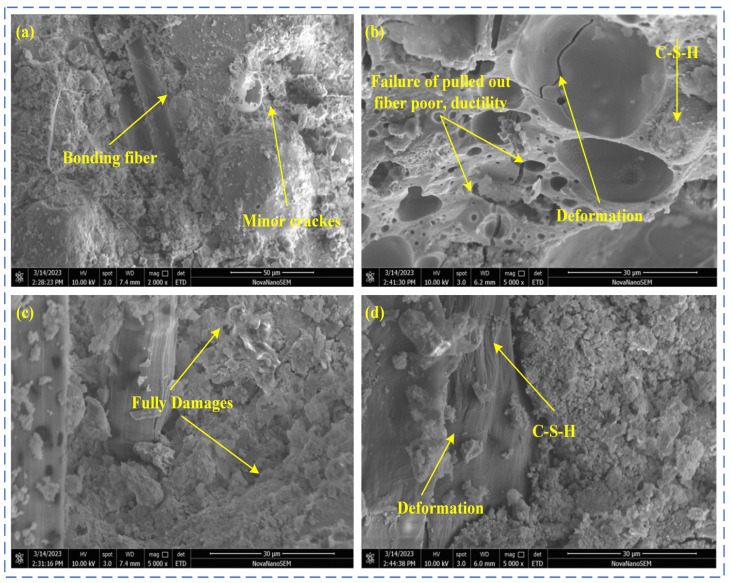
Microstructure of YRS-ECC matrix under different curing methods. (**a**) Standard curing (**b**) steam curing, (**c**) sprinkling curing, (**d**) natural curing.

**Figure 15:**
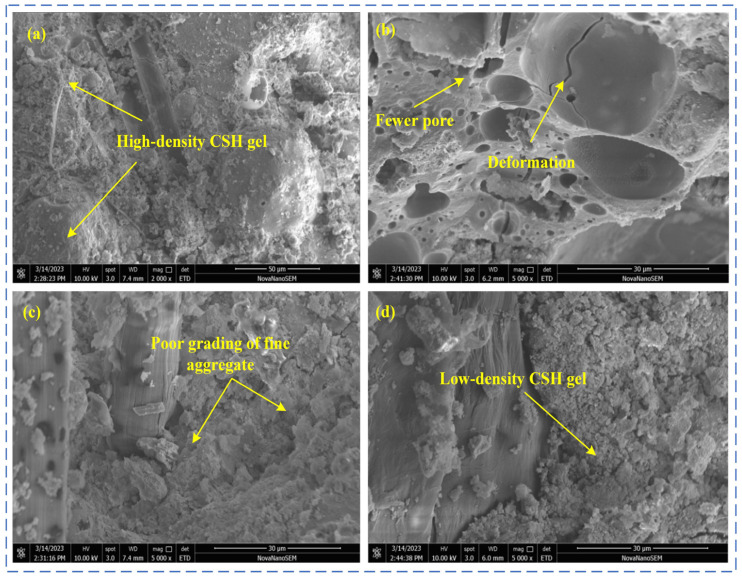
Microstructure of YRS-ECC fiber–matrix interface under different curing methods. (**a**) Standard curing, (**b**) steam curing, (**c**) sprinkling curing, (**d**) natural curing.

The YRS-ECC under four curing methods at 90 days of age was selected, and its microstructure was observed through SEM experiments. The microstructure of the YRS-ECC matrix under different curing methods is shown in Figure 14. The micro-morphology of the ECC fiber–matrix interface of the YRS under different curing methods is shown in Figure 15.

Figure 14 reveals that the matrix microstructure of the YRS-ECC under four curing methods: After 90 days of standard curing (Figure 14a), the C-S-H is massive, and the fly ash is firmly embedded in the matrix. The amount of C-S-H gel produced by secondary hydration on the surface is extensive, almost covering it, indicating a high internal hydration degree of the matrix. Under steam curing (Figure 14b), hemispherical pits left by fly ash fall-off are clearly visible. Compared to the other three curing methods, there are many high-density C-S-H gels (HD C-S-H). The hydration rate of cement is accelerated at higher temperatures, increasing the aggregation rate of hydration product particles and altering the packing density of the particle structure, thereby increasing the crystallization tendency of the C-S-H gel. Wide microcracks are caused by delayed AFt generation, which occurs when expansive AFt forms in large quantities inside the hardened concrete as the cement hydration cools to room temperature, resulting in stress that leads to cracks. Additionally, many small holes are present due to the destruction of the internal pore structure caused by the two-phase transformation process of water and gas under high-temperature curing. The high temperature accelerates the migration of surface water into the concrete, leaving numerous connected holes, and internal water transforms into gas, forming bubbles that leave holes when they burst inside [52]. Under sprinkler curing (Figure 14c), the surface of fly ash is covered with small granular C-S-H gel, and the secondary hydration reaction on the surface is sufficient. Long microcracks appeared in the pores left by fiber pull-out due to the high interfacial chemical bonding strength between the fiber and the matrix, which led to the fracture of the nearby matrix when the fiber was pulled out by the load. A small number of needle-like AFt was also generated on the right side of the channel. Lastly, under natural curing (Figure 14d), there are also many C-S-H gels, but they are flocculent C-S-H (I) gels with low density. This is because, when the water inside the specimen is consumed by cement hydration, natural curing cannot provide additional water for the hydration reaction of the specimen. Consequently, the low degree of hydration leads to low-density hydration products and relatively low compressive strength.

Figure 15 shows the microstructure of the fiber–matrix interface of the YRS-ECC under four curing methods. In Figure 15a, many small granular C-S-H gels are distributed on the surface of the fibers, with almost no gap between the fibers and the matrix. These factors contribute to the difficulty of smoothly pulling out the fibers from the matrix, causing significant ductility deterioration of the YRS-ECC after 90 days of standard curing. In Figure 15b, a gap between the fiber and the matrix is observed under steam curing. This gap is due to the high pozzolanic activity of fly ash under steam curing, where the generated acidic oxide reacts with Ca(OH)_2_ attached to the fiber surface. Studies have shown that the bond strength between the fiber and the matrix is dominated by the content of Ca(OH)_2_ at the interface. The high temperature of steam curing promotes the consumption of more Ca(OH)_2_ by fly ash, reducing the bond strength between the fiber and the matrix. This explains why the concrete strength remains high, but the ductility does not decrease significantly after steam curing, with the 90-day ultimate tensile strain still greater than 3%. In Figure 15c, after 90 days of sprinkler curing, the fibers were torn during the pull-out process after debonding, resulting in serious fiber damage. This damage explains the short strain-hardening process and poor ductility of the specimens under sprinkler curing. In Figure 15d, a micro-gap between the fiber and the matrix is observed, and the integrity of the fiber remains good after being pulled out. Therefore, the specimen can exhibit pseudo-strain hardening under natural curing, resulting in relatively high ultimate tensile strain.

#### 3.3.2. MIP Experiments

**Figure 16:**
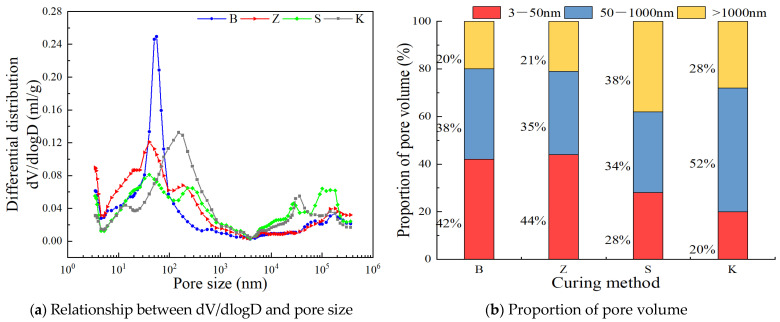
Comparative study of pore characteristics of YRS-ECC under different curing methods.

The YRS-ECC under different curing methods at 90 d age was selected for MIP experiments. According to the results of mercury injection test, the pore size is divided into three intervals: 3~50 nm, 50~1000 nm, and >1000 nm. The relationship between dV/dlogD and pore size of YRS-ECC under different curing methods is shown in Figure 16a. The pore volume of YRS-ECC under different curing methods is shown in Figure 16b.

The porosity and average pore size of the specimen under natural curing are the largest, at 32.86% and 51.69 nm, respectively, with only 20% of the pores having a diameter less than 50 nm. The porosity and average pore size of sprinkler curing are only slightly better than those of natural curing. The average pore size under steam curing is the smallest, with the highest proportion of pores less than 50 nm, reaching 44%. The total porosity under steam curing is greater than that under standard curing. This is because steam curing can accelerate the hydration of cement, generating more C-S-H gel to fill the pores, increasing the number of gel pores with smaller pore sizes, and leading to the coarsening of the overall pore structure. The porosity of the sample under standard curing is the smallest, only 27.25%, and the pore volume distribution range is similar to that under steam curing. This is because standard curing can provide a suitable temperature and continuous humidity, resulting in a stable hydration process of cement.

## 4. Mechanical Properties of YRS-ECC and Traditional Sand ECC

Under standard curing conditions, compressive and flexural tests, uniaxial tensile test, and four-point bending tests were carried out on two kinds of test blocks with age of 28 days to evaluate whether the YRS can meet the mechanical performance requirements in engineering applications after replacing quartz sand.

### 4.1. Compressive and Flexural Strength

**Figure 17:**
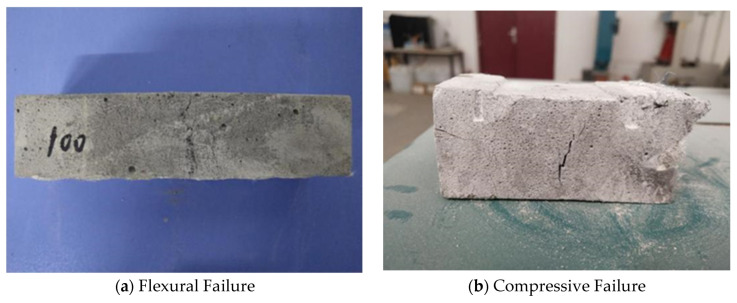
Failure modes of flexural and compressive specimens.

The flexural tests of the YRS-ECC test specimens and traditional sand ECC specimens block were carried out. No brittle fracture occurred in the flexural specimens, and the flexural failure mode is shown in Figure 17a. A main crack and many small cracks appeared on the tensile surface of the specimens, which still maintained good integrity. There is no brittle failure in the compressive test block, and the compressive failure mode is shown in Figure 17b. It can be seen from the figure that only some small cracks appear on the surface when the specimen is destroyed, and the test block is depressed downward. The deformation is large but the overall integrity is still good. The compressive strength and flexural strength of traditional ECC are 27.4 MPa and 12.2 MPa, respectively. The compressive strength and flexural strength of YRS-ECC are 26.4 MPa and 11.5 MPa, respectively. Since the particle size of the YRS is smaller than that of quartz sand, when the YRS is used instead of quartz sand, it is equivalent to reducing the particle size of fine aggregate in ECC, resulting in a decrease in strength. However, the experimental results show that the strength difference between YRS-ECC and traditional sand ECC is not significant.

### 4.2. Uniaxial Tensile Property

**Figure 18:**
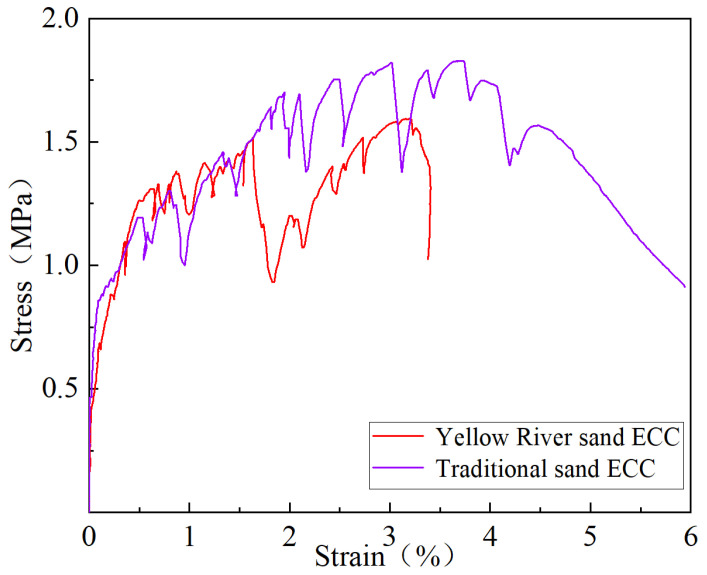
The tensile stress–strain curves of YRS-ECC and traditional sand ECC.

Based on the uniaxial tensile test, the average values of tensile performance parameters such as ultimate tensile strength and ultimate tensile strain of the specimens are calculated. The tensile performance parameters of the two ECCs are shown in Table 7. The tensile stress–strain curves of the two ECCs are shown in Figure 18.

When the quartz sand was completely replaced with the YRS with a 100% substitution rate, it was observed that the initial crack strength was only reduced by 0.1 MPa. Specifically, the initial crack strain of traditional sand ECC is 0.05%, while the initial crack strain of YRS-ECC is 0.02%. Although the initial cracking strain decreased, the ultimate tensile strength of the YRS-ECC increased from 1.60 MPa to 1.83 MPa, and the ultimate tensile strain increased from 3.20% to 3.71%. These results show that it is feasible to completely replace quartz sand with YRS as a fine aggregate of ECC, and some performance indexes are improved. These data support the effectiveness of YRS in ECC applications, especially in terms of ultimate tensile strength and strain.

### 4.3. Four-Point Bending Test 

**Figure 19:**
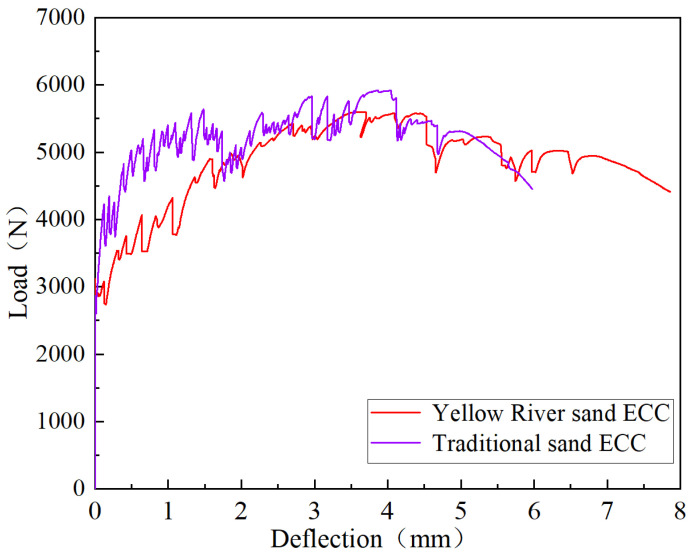
Bending load–deflection curves of YRS-ECC and traditional sand ECC.

Through the load–deflection curve in the four-point bending test, the displacement characteristic points of the YRS-ECC can be determined. These characteristic points include initial cracking load, initial cracking deflection, ultimate bending load, and ultimate bending deflection. The average bending performance parameters of the YRS-ECC and the traditional sand ECC are calculated. The results of detailed bending performance parameters are shown in Table 8. The bending load–deflection curves of the YRS-ECC and the traditional sand ECC are shown in Figure 19. In the four-point bending performance test, the ultimate bending load and deflection of YRS-ECC were 5.7% and 9.4% higher than those of traditional sand ECC, respectively. This result is consistent with the trend observed in the uniaxial tensile test, which further verifies the improvement effect of the YRS on the flexural properties of ECC. This shows that the use of YRS with a 100% substitution rate as the fine aggregate of ECC can significantly improve the ductility and bending resistance of the material.

### 4.4. Five-Dimensional Evaluation Diagram

**Figure 20:**
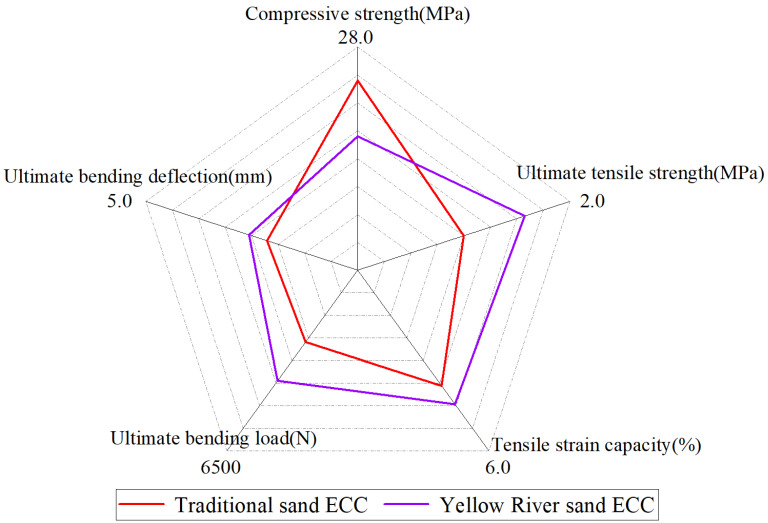
Five-dimensional evaluation diagram of Yellow River sand ECC and traditional sand ECC.

Combined with the mechanical properties test results of the YRS-ECC and the traditional sand ECC, five indicators were selected: compressive strength, ultimate tensile strength, ultimate tensile strain, ultimate bending load, and ultimate bending deflection. The five-dimensional evaluation method [53] was used for comparative analysis. The five-dimensional evaluation diagram of ECC under different YRS replacement rates is shown in Figure 20.

It can be seen from Figure 20 that the strength and deformation capacity of YRS-ECC show a good balance. This result was verified by a four-point bending performance test, indicating that the performance of YRS-ECC in ultimate bending load and deflection is more balanced. In addition, from the perspective of engineering practice, the use of 100% YRS to completely replace quartz sand can not only maximize the use of YRS resources but also bring more economic and environmental benefits. Therefore, it is a feasible optimization scheme to apply YRS as a full alternative material to ECC.

## 5. Conclusions

This study investigates the use of YRS as a replacement for natural quartz sand in the preparation of ECC. ECC specimens with 100% YRS replacement with quartz were evaluated under different curing methods including natural curing, steam curing, standard curing, and sprinkler curing. Comprehensive tests, including flexural, compressive, uniaxial tensile, and four-point flexural tests, were conducted. Additionally, SEM and MIP tests explored the microscopic mechanisms influencing macroscopic mechanical properties. Below are the key findings of this study.

(1) Steam curing significantly enhances the compressive strength of YRS-ECC, exhibiting peak strengths at 14 days, 28 days, and 90 days. Natural curing and sprinkler curing initially show higher early strengths compared to standard curing, but their strength gains decelerate between 28 days and 90 days, lagging behind standard curing.
(2) ECC specimens made with YRS achieve the highest ductility under natural curing at 14 days, 28 days, and 90 days, with the ultimate tensile strain still exceeding 4% at 90 days. Steam curing maintains a 3% strain at 90 days. Specimens under standard curing and sprinkler curing fail to meet the 3% strain requirement beyond 14 days. Natural curing results in fine cracks post-failure, exhibiting a superior crack index compared to other curing methods.
(3) Ductility decreases as specimens age from 14 days to 90 days across all curing methods. Natural curing shows the least decline, achieving a maximum ultimate flexural deflection of 4.42 mm at 90 days. Steam curing achieves the highest ultimate flexural load of 5004.82 N, with the largest toughness ratio at 90 days among all methods.
(4) Steam curing promotes the formation of dense C-S-H gel with micro-gaps that facilitate fiber pull-out. Standard and sprinkler curing methods generate extensive C-S-H gel but cause significant fiber damage upon pull-out. Natural curing produces flocculent C-S-H gel, maintaining better fiber integrity after pull-out. Additionally, natural curing exhibits the highest porosity (32.86%) and average pore size (51.69 nm). Steam curing results in the smallest average pore size, with a notable fraction of pores less than 50 nm (44%).
(5) Based on compressive strength, uniaxial tensile, four-point flexural tests, and microscopic analysis, natural curing emerges as the most suitable maintenance method for 100% YRS-ECC in practical engineering applications.
(6) Under the standard curing conditions, the flexural strength, uniaxial tensile test and four-point bending test were carried out on the two test blocks of 14 days. The results show that the ultimate bending load and deflection of YRS-ECC are 5.7% and 9.4% higher than those of traditional sand ECC, respectively, and the ultimate tensile strength and strain are also improved. These results show that the YRS-ECC has obvious advantages in improving the ductility and bending properties of the material, thus verifying its effectiveness in ECC applications.

## Figures and Tables

**Table 1:** PVA fiber technical indicators.

Diameter (µm)	Length (mm)	Tensile Strength (MPa)	Elastic Modulus (GPa)	Elongation at Break (%)	Density (g·cm^−3^)
40	12	1560	41	6.5	1.3

**Table 2:** Main technical indexes of YRS.

Type of Fine Aggregate	Apparent Density/kg·m^−3^	Bulk Density/kg·m^−3^	Water Absorption at Saturated Surface-Dry Basis/%	Specific Surface Area/m^2^·g^−1^
Quartz sand	2650	1675	0.2	0.073
YRS	2647	1418	1.1	0.435

**Table 3:** The mix proportion of YRS-ECC and traditional sand ECC (kg/m³).

Types of ECC	Water	Fly Ash	Cement	Quartz Sand	YRS	Water Reducer	Thickener	PVA Fiber
YRS-ECC	410	585	585	0	700	3.5	1.8	19
Traditional sand ECC	410	585	585	700	0	3.5	1.8	19

**Table 4:** Grouping of mechanical properties test.

Performance Index	Peer Group	Specimen Size	Quantity
compressive and flexural strength	B14, B28, B90	160 mm × 40 mm × 40 mm	39
	Z14, Z28, Z90		
	S14, S28, S90		
	K14, K28, K90 B’28		
uniaxial tensile property	B14, B28, B90	330 mm × 60 mm × 13 mm	78
	Z14, Z28, Z90		
	S14, S28, S90		
	K14, K28, K90 B’28		
four-point bending test	B14, B28, B90	160 mm × 40 mm × 40 mm	39
	Z14, Z28, Z90		
	S14, S28, S90		
	K14, K28, K90 B’28		

**Table 5:** The percentage change in compressive strength with different curing methods.

Serial Number	Curing Age	Flexural Strength (MPa)	Variation Trend	Compressive Strength (MPa)	Variation Trend
B14	14 d	9.9	-	22.9	-
Z14		14.8	49.50%	33.0	44.10%
S14		11.8	19.20%	27.7	20.96%
K14		11.7	18.19%	27.3	19.21%
B28	28 d	11.5	-	26.4	-
Z28		15.7	36.52%	34.9	32.20%
S28		12.5	8.70%	29.3	10.98%
K28		13.2	14.78%	30.8	16.67%
B90	90 d	14.0	-	32.7	-
Z90		17.6	25.71%	38.7	18.35%
S90		13.4	−4.29%	31.6	−3.36%
K90		13.3	−5.00%	31.1	−4.93%

**Table 6:** The percentage change in compressive strength under different curing time.

Serial Number	Curing Method	Flexural Strength (MPa)	Variation Trend	Compressive Strength (MPa)	Variation Trend
B14	Standard curing	9.9	-	22.9	-
B28		11.5	16.16%	26.4	15.28%
B90		14.0	21.74%	32.7	23.86%
Z14	Steam curing	14.8	-	33.0	-
Z28		15.7	6.08%	34.9	5.76%
Z90		17.6	12.10%	38.7	10.89%
S14	Sprinkler curing	11.8	-	27.7	-
S28		12.5	5.93%	29.3	5.78%
S90		13.4	7.20%	31.6	7.85%
K14	Natural curing	11.6	-	27.3	-
K28		13.2	13.80%	30.8	12.82%
K90		13.3	0.75%	31.1	0.97%

**Table 7:** Tensile performance parameters of YRS-ECC and quartz sand ECC.

Types of Sand	Initial Crack Strength σ_fc_ (MPa)	Initial Cracking Strain ε_fc_ (%)	Ultimate Tensile Strength σ_tu_ (MPa)	Tensile Strain Capacity ε_tu_ (%)
Quartz sand	0.48	0.05	1.60	3.20
Yellow River sand	0.47	0.02	1.83	3.71

**Table 8:** Bending performance parameters of YRS-ECC and traditional sand ECC.

Types of Sand	Initial Crack Load F_fc_ (N)	Initial Crack Deflection δ_fc_ (mm)	Ultimate Bending Load F_tu_ (N)	Ultimate Bending Deflection δ_tu_ (mm)
Quartz sand	3133.13	0.01	5596.81	3.71
Yellow River sand	2867.15	0.02	5917.73	4.05

## Data Availability

We would like to declare that the data employed and analyzed during the current study will be available from the corresponding author upon request, and we are committed to providing access to our data to ensure the transparency and reproducibility of our findings.
